# On *Hypolycaena* from Maluku, Indonesia, including the first description of male *Hypolycaena asahi* (Lepidoptera, Lycaenidae)

**DOI:** 10.3897/zookeys.115.1406

**Published:** 2011-07-05

**Authors:** Alan Cassidy, Andrew Rawlins

**Affiliations:** 1 18 Woodhurst Road, Maidenhead, Berkshire, SL6 8TF, England; 2 392 Maidstone Road, Rainham, Kent, ME8 0JA, England

**Keywords:** *Hypolycaena*, *asahi*, *danis*, *dictaea*, *erylus*, *phorbas*, *pigres*, *silo*, *sipylus*, Indonesia, Maluku, Lepidoptera, Lycaenidae

## Abstract

The taxonomy and distribution of the five species of *Hypolycaena* in Maluku are discussed and new locality records given. Corrections are made to the published taxonomy and distribution of *Hypolycaena phorbas* (Fabricius, 1793). This clarification enables a better understanding of the biogeography of the genus. *Hypolycaena asahi* Okubo, 2007, was originally described from a single female from Ambon and is here recorded from Seram. The male is described for the first time.

## Introduction

The Indonesian provinces of North Maluku and Maluku consist of numerous islands, yet their butterfly fauna remains less well described than those of the principal surrounding areas of the Philippines, Sulawesi and New Guinea. Vane-Wright and Peggie (1994) comment that, geologically, the northern islands of Halmahera, Ternate, Morotai and Bacan form a complex of land areas variously related to New Guinea, while the Buru, Ambon, Seram arc is related to North-West Australia. The Sula islands of Taliabu, Mangole and Sanana, in the west of Maluku were included faunistically in the “Sulawesi region” by Vane-Wright and de Jong (2003), while Burrett et al. (1991) link Sula geologically with Banggai and Obi. The islands of the Aru group in the south east of Maluku share the continental shelf of, and are faunistically close to, the New Guinea mainland.

Thus Maluku *sensu lato* remains an area of immense biogeographical interest, with the largest of its islands forming the northeasterly part of Vane-Wright’s “Wallacea”: the land between the Sunda and Sahul shelves. To facilitate testing of biogeographical hypotheses, it is important that the taxonomy and distributional data of all butterfly families represented in Maluku is accurate and as comprehensive as possible. The extensive lycaenid fauna is perhaps the least understood.

The genus *Hypolycaena* C. & R. Felder, 1862, (Lycaenidae, Theclinae, Hypolycaenini) consists of about 25 species in the Indo-Australian region as well as about 20 species in Africa. Fiedler (1992) included *Chliaria* Moore, 1884, and *Zeltus* de Nicéville, 1890, within *Hypolycaena* whilst Eliot (1992), retained these as separate genera in his subtribe Hypolycaeniti. *Hypolycaena asahi* was described by Okubo, in 2007, from a single female specimen. The male is described here for the first time and enables its relationship with other species of the genus to be more closely determined.

This paper is primarily concerned with *Hypolycaena* species in Maluku. However, it is necessary to discuss in some detail the taxonomy and wider distribution of *Hypolycaena phorbas* (Fabricius, 1793) and its allies. These taxa form a species group in which the males exhibit a large circular dark brand of apparently normal scales on the upperside of the forewing and in which the early stages are polyphagous and strongly myrmecophilic (Fiedler, 1992). This study will confirm the identity of the taxon found on Aru Islands and also clarify D’Abrera’s record of *Hypolycaena erasmus* Grose-Smith, 1900, in Halmahera. A more accurate understanding of the taxonomy and distribution of the *phorbas* species group will in turn lead to a better understanding of the biogeography of the Papua mainland and the islands to the East and West of it.

Note that the frequent references to Parsons and D’Abrera refer to Parsons (1998) and D’Abrera (1978).

## Geopolitical terminology

The Indonesian western half of the Island of New Guinea and its associated offshore islands, which has previously been known as Irian Jaya, now consists of two provinces: Papua and West Papua. However “Papua” has also been used to denote this whole area. For simplicity we will use the term “Papua mainland” to describe the whole area excluding offshore islands.

## Equipment and methods

The preserved material forming the basis of this study is primarily that of the collections of the Natural History Museum London (BMNH) and of the second author. Where their reliability is assured, other distributional data have been accepted in correspondence from curators of other private collections.

Male genitalia were prepared by soaking in 0.1N potassium hydroxide solution for 24 hours at room temperature prior to dissection. Micro-photography of the genitalia, while suspended in 80% Iso-Propanol, was with an AIGO GE-5 digital microscope and the images were subsequently processed using Helicon-Focus 5.0 software (Helicon Soft Ltd. 2010) to enhance depth of field.

All photographs of preserved adult specimens, except those kindly provided by Mr. Yusuke Takanami, were taken using a Nikon D80 digital SLR camera fitted with a Micro-Nikkor 60mm macro lens. The photographic images presented were post-processed for exposure compensation, cropping, resizing and sharpening using Adobe Photoshop Elements 6.0. The scale on photographs represents multiples of 5mm.

***Hypolycaena asahi* Okubo, 2007**

♂Figs 1,2, 10, Fig. 9 genitalia; ♀ Figs 3–8, 10.

**Figures 1–8.**
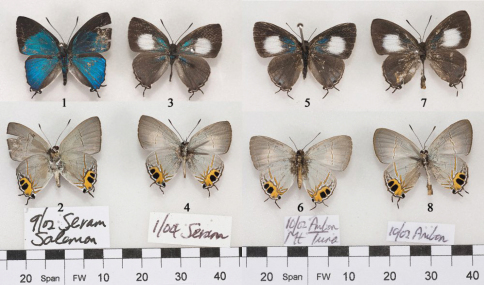
*Hypolycaena asahi*, top row recto, bottom row verso. **1, 2** male, Seram **3, 4** female, Seram **5–8** females, Ambon.

The holotype female was captured in March 2000. The type location was given by Okubo as “Mt. Tuna, *ca* 900m, Ambon Island, North Moluccas [sic], Indonesia”. Four further specimens of *Hypolycaena* were captured in Central Maluku in 2002 and 2004, comprising three females and one male. These four specimens are illustrated in Figs 1–8. Two of the three female specimens are from the *Hypolycaena asahi* type location in Ambon, while a male and female are recorded for the first time from Salemon in Seram.

The external morphology of all of these new females is inseparable from that of the *Hypolycaena asahi* holotype, and their identification as examples of *Hypolycaena asahi* is assured. We propose the hypothesis that the male specimen is also *Hypolycaena asahi* because of its underside markings and its sympatry with the aforementioned female from Seram.

**♂ Upperside.** Forewing length 13mm. Both fore and hindwings metallic blue with dark borders. The forewing black border about 1mm wide at the tornus but rapidly widening along the termen to meet the costa at its mid-point, then running down to the base above vein 12, but not quite entering the cell. The forewing also with basal swelling of veins 2, 3 and 4 with a faint brand of seemingly normal (not androconial) scales surrounding these swollen veins. The hindwing black between veins 7 and 8, and with a dark grey dorsal border in spaces 1 and 1a. In space 1a a small black tornal lobe with a white marginal streak. Filamentous white-tipped black tails at veins 1b and 2, 2mm and 3mm long respectively.

**Underside.** No significant differences exist between the undersides of the females from Ambon and those of both sexes from Seram.

**♂ Genitalia.** Saccus short, bluntly pointed. Brachia long and tapering to a fine point, with a broad elbow and a pronounced lobe at the proximal junction with the tegumen. Valvae short, broad and conjoined basally, tapering distally with the apex rounded and the inner margins finely serrate. Aedeagus medium length, the sub-zonal portion shorter that the supra-zonal portion.

**Figure 9.**
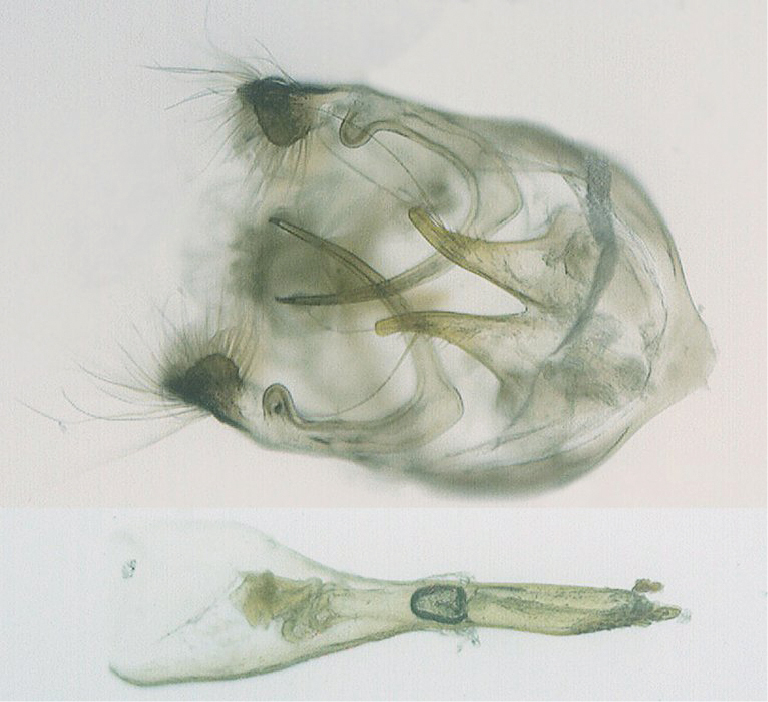
Male genitalia of *Hypolycaena asahi* Okubo, 2007, from Seram, showing ventral view of armature and lateral view of aedeagus.

**Figure 10.**
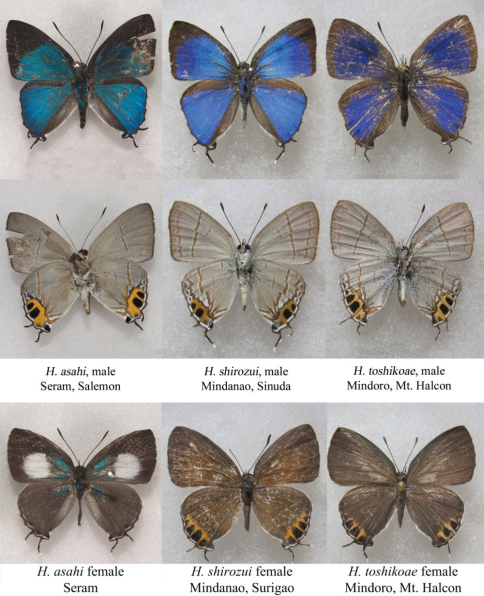
*Hypolycaena asahi* Okubo, compared with *Hypolycaena shirozui* Hayashi and *Hypolycaena toshikoae* Hayashi from the Philippines. Philippine photos courtesy of Mr. Yusuke Takanami.

**Remarks.** The early stages are unknown. The females of *Hypolycaena asahi* (Figs 3–8) from both locations show varying amounts of basal blue scaling not evident in the holotype. Otherwise, they conform closely to Okubo’s description.

Okubo notes the similarity between this species and two allied species from the Philippines: *Hypolycaena shirozui* (Hayashi, 1981) and *Hypolycaena toshikoae* Hayashi, 1984 (Fig. 10). In all three species the underside hindwing tornal orange area extends into space 3 and the sub-marginal black spot in space 3 is much larger and darker than in space 4. On the underside in both sexes, *asahi* shows a much more marked dislocation of the post-discal band than in *shirozui*, while this dislocation is absent in *toshikoae.* On the male upperside, the apical black border in *asahi* is much broader than in either of the other two species, most notably in spaces 2, 3 and 4. On the female upperside, neither *Hypolycaena shirozui* nor *Hypolycaena toshikoae* exhibits the white forewing discal patch of *Hypolycaena asahi*. Both Philippine species have orange inwardly surrounding the hindwing sub-marginal lunules, which is absent in *Hypolycaena asahi* from both Seram and Ambon.

In the male genitalia, *Hypolycaena asahi* is distinguished from these other species by the more elongate valvae, which have more rounded apices, and by the shorter suprazonal portion of the aedeagus.

## Taxonomy and distribution of Other Hypolycaena species in Maluku

In addition to *Hypolycaena asahi* there are four other *Hypolycaena* species in Maluku. Fiedler, 1992, pointed out that two of these, *Hypolycaena phorbas* and *Hypolycaena erylus* (Godart, [1824]) shared characteristics of larval polyphagy and strong mymecophily and referred to them as the *phorbas* species group. Adult males in this group also have a large circular black brand on the upperside of the forewing. It group includes the taxa *erylus*, *phorbas*, *dictaea* C. & R. Felder, 1865, (**TL:** Waigeo) and *periphorbas* Butler, 1882 (**TL:** New Britain), all of which Parsons treated as separate species.

D’Abrera treated *Hypolycaena erasmus* (**TL:** New Ireland) as a valid species name and *moutoni* Ribbe, 1899 (**TL:** Duke of York Island) as a subspecies of *Hypolycaena erylus*. Parsons synonymised *erasmus* and *moutoni* with *periphorbas*, giving *periphorbas* species status. D’Abrera had listed *periphorbas* as a subspecies of *phorbas*. Parsons noted that *Hypolycaena periphorbas* is restricted to the Bismarck Archipelago. We accept Parsons’ synonymies.

D’Abrera illustrated the upperside of a female *Hypolycaena* specimen from Halmahera which he labelled “*Hypolycaena erasmus* subsp.?” We examined this specimen in BMNH which carries a label stating: “Specimen photographed by B. D’Abrera, 1970”. The specimen is clearly *Hypolycaena erylus thyrius* and matches other *thyrius* specimens in the same column. This specimen is shown here in Figs 29 and 30 which should be compared with Figs 17 and 18 from Bacan.

Therefore we conclude there is no record of *Hypolycaena periphorbas* (= *Hypolycaena erasmus*) occurring in Maluku. In the Maluku fauna, *Hypolycaena phorbas* can be readily distinguished from *Hypolycaena erylus* by its broader dark forewing margin in the male (Fig. 31) and its white forewing patch in the female (Figs 33, 35).

***Hypolycaena erylus*** (Godart, [1824]) (Type Locality (TL): “De Java”)

This species ranges from India to Indonesia, the Philippines and New Guinea. Within Maluku *Hypolycaena erylus* is known from N. Maluku, the Sula Islands and there are a few specimens in BMNH (The Natural History Museum in London) from S.E. Maluku (see below).

**Figures 11–14.**
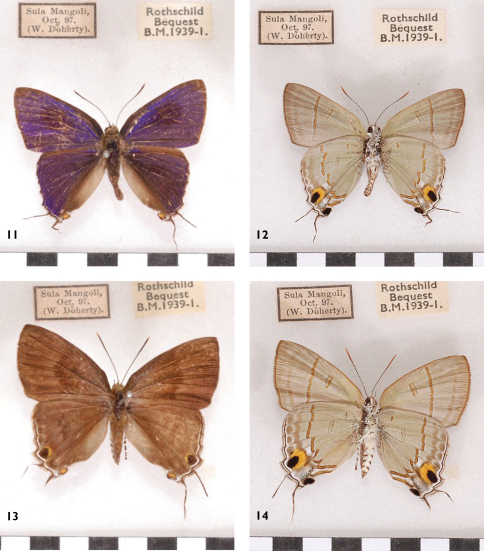
*Hypolycaena erylus gamatius*, Sula Islands, Mangole, l. to r.: ♂ Up, Un, ♀ Up, Un.

***Hypolycaena erylus gamatius*** Fruhstorfer, [1912] (TL: Toli-Toli, N. Sulawesi). Figs 11–14.

*Hypolycaena erylus gamatius* Fruhstorfer has been recorded from Mangole (Vane-Wright & de Jong, 2003) in the Sula Islands.

Specimens received by the second author from Taliabu (Jorjoga - 1♂, 1♀, ii/2001, 1♂, 1♀ x/2001, 1♂ v/2002, 1♂ iii/2004) represent a new island record. We also add the islands of Muna (1♂, 1♀ iii/2008) and Timpaus (5♂♂, 7♀♀ vii/2006) as new locality records, although not in Maluku Province.

**Figures 15-18.**
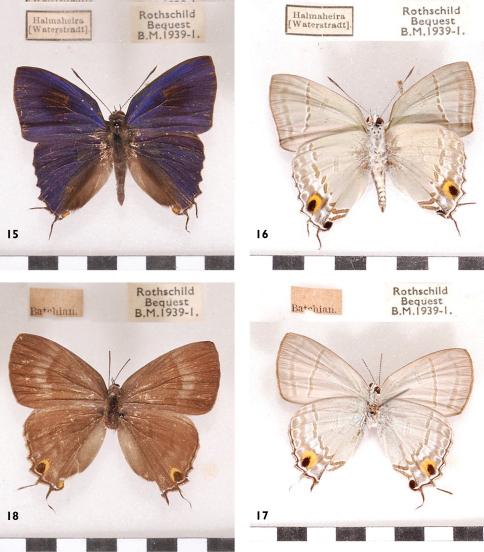
*Hypolycaena erylus thyrius*, l. to r.: ♂ Halmahera Up, Un, ♀ Bacan Up, Un.

***Hypolycaena erylus thyrius*** Fruhstorfer, [1912] (TL: Halmahera). Figs 15–18.

**Figures 19–22.**
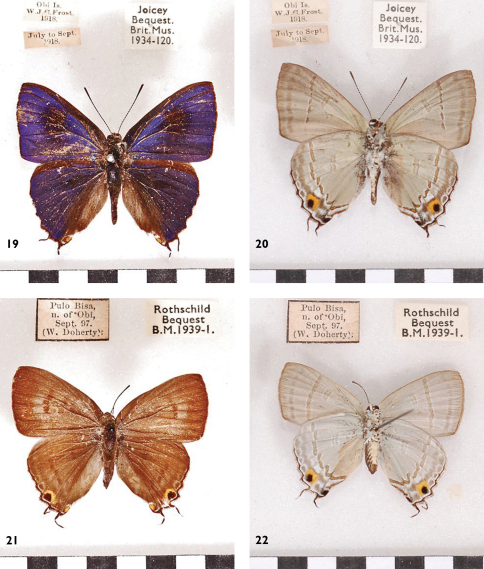
*Hypolycaena erylus thyrius* *(= pigres)*,Obi, l. to r.: ♂ Up, Un, ♀ Up, Un.

= *Hypolycaena erylus pigres* Fruhstorfer, [1912] syn. nov. (TL: Obi). Figs 19–22.

= *Hypolycaena erasmus* ssp; D’Abrera 1978: 304. [Misidentification].

Fruhstorfer described *Hypolycaena erylus thyrius* from “Halmaheira, Batjan” but listed only a single female type. This taxon is known from Halmahera, Ternate and Bacan. To this we add Morotai (Daeo – 2♂♂ 3/vi/1992, 1♂ ii/1998, 1♂ iii/1998, 2♀♀ iv/2004, 1♀ 25/viii/1995, 1♀ 2/xi/1995), Buho-Buho (1♂ 8/xii/90) and Kasiruta (1♂ iv/2003).

In the same paper Fruhstorfer described *Hypolycaena erylus pigres* from Obi, based on a series of eight males. Having examined the *pigres* and *thyrius* holotypes, as well as a long series of Obi and Halmahera specimens, we can not see any clear differences between the two taxa and therefore consider *pigres* to be a synonym of *thyrius*, which appears earlier in Fruhstorfer’s work.

**Figures 23-26.**
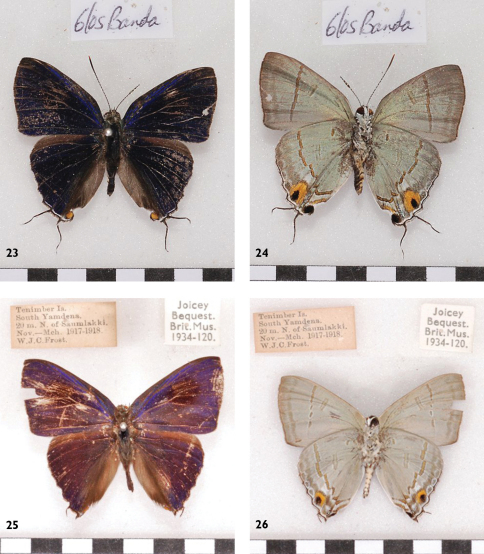
l. to r.: *Hypolycaena erylus* ssp ♂ Banda Up, Un, ♂ Tanimbar Up, Un

**Figures 27-30.**
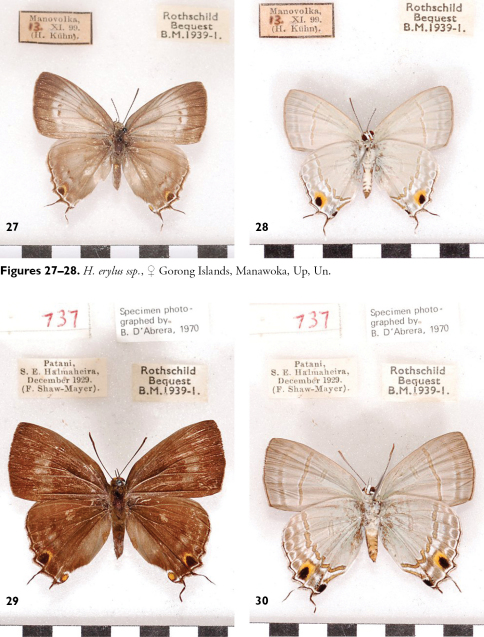
“*Hypolycaena erasmus* ssp” D’Abrera, 1977: 304 recte *Hypolycaena erylus thyrius* ♀, Halmahera.

**Figures 31-34.**
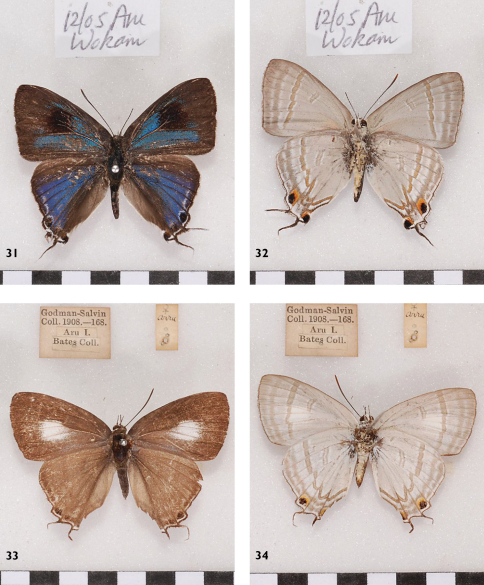
*Hypolycaena phorbas silo*, l. to r.: Aru, Wokam, ♂ Up, Un, Aru, ♀ Up, Un.

***Hypolycaena erylus*** *incertae sedis.* Figs 23–28.

Although *Hypolycaena erylus* is widespread in the South-East Asian islands and into New Guinea, material from South and South-East Maluku is scarce. We have seen a single male from Banda and BMNH has three males of *Hypolycaena erylus* from Tanimbar (20 miles north of Saumlaki, Yamdena - 1917–1918, Frost). These are all difficult to assign to a particular named subspecies (the males of the different subspecies tend to be fairly similar whilst the females vary more). See Figs 23–26.

In addition BMNH holds one female from Manawoka Island (label reads: Manovolka. 13.xi.(18)99. H. Kühn) in the Gorong Islands, which is unlike any other subspecies, having extensive pale areas on the upperside - especially the forewing - and may represent a new subspecies. See Figs 27, 28.

We await further confirmatory material before naming any further subspecies based on these few specimens.

***Hypolycaena phorbas***(Fabricius, 1793) (TL: “Ins. Papuanae”). Figs 31–42.

Parsons reviewed *Hypolycaena phorbas* from Papua New Guinea stating that two subspecies occur there: *silo* Frustorfer, [1912] and *infumata* Fruhstorfer, 1910. He synonymised *latostrigatus* van Eecke, 1915, *pseudophorbas* Fruhstorfer, [1914], *phorbanta* Rothschild, 1916 and *walteri* Fruhstorfer, [1916a] with *silo.* He gave the range of *phorbas* as Waigeo, Biak, Roon, mainland New Guinea, various outlying islands and Australia.

D’Abrera and Seitz, 1926, considered Felder’s taxon *dictaea* to be a subspecies of *phorbas* found on Waigeo only, whereas Parsons “provisionally treated” *dictaea* as a separate species and stated the range to include Aru, Waigeo, mainland New Guinea and its varying outlying islands as far south east as Australia. He went on to specify a number of island localities.

Therefore according to Parsons, two “species”, *Hypolycaena phorbas* and *Hypolycaena dictaea*, occur on Waigeo as well as mainland New Guinea. However, we consider that *phorbas* and *dictaea* are conspecific and that only one subspecies, *Hypolycaena phorbas silo*, occurs in political Maluku, on Aru, its type locality and on mainland Papua. As Aru is separated from mainland New Guinea only by shallow water, and may well have been directly connected at the surface during the last glaciation, it can be regarded biogeographically as part of Papua.

***Hypolycaena phorbas dictaea*** C. & R. Felder, 1865 (TL: Waigeo) Figs 37, 38, 41, 42.

Parsons did not locate the holotype female of *dictaea* although it is deposited in BMNH. We have examined this specimen along with a series of female specimens in BMNH from Waigeo and it is clear that they all have undersides that are significantly paler in ground colour and weaker in the post discal striae than the underside of the holotype female of *silo* (also at BMNH).

We have also examined females, whose undersides are dark and therefore match that of the *silo* holotype, from Papua mainland, Aru, Roon, Biak and Yapen in BMNH and the collection of the 2nd author.

Additionally, we have studied a series of males from Waigeo. Unlike the females, the Waigeo males’ undersides vary, ranging from the paleness of the holotype female *dictaea* to the much darker underside of the holotype female of *silo*.

***Hypolycaena phorbas silo*** Fruhstorfer, [1912] (TL: “Neu-Guinea Fr. Wilh. Hafen.”) Figs 31–36, 39, 40.

**Figures 35-38.**
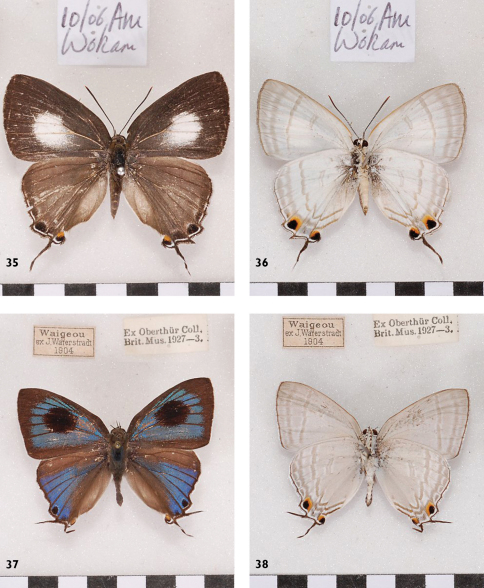
*Hypolycaena phorbas silo* Aru, Wokam, ♀; *Hypolycaena phorbas dictaea* Up, Un, Waigeo, ♂ Up, Un.

Within Maluku this subspecies is only found on Aru Islands. We have examined five males and five females from Aru. All display the typical *silo* phenotype with the exception of one female from Wokam (Figs 35, 36), which has a slightly lighter underside than the other four.

We present new records of this subspecies from Wokam Island in the Aru group, (1♂ xi/2004, 1♂ xii/2005, 1♀ x/2006).

### Other *phorbas* material examined, from outside Maluku.

Undersides of series of males from Batanta, Papua mainland, Aru, Roon, Biak and Yapen are all of the darker form matching the holotype female *silo* underside. The three males and three females from Misool in BMNH all have the paler undersides matching the *dictaea* type specimen. There is one male from Salawati in BMNH whose underside is of this form.

We have also examined male genitalia from specimens from Waigeo (Fig. 75), Aru (Fig. 76), Batanta (Fig. 77), Yapen (Fig. 78) and Papua mainland (Fig. 79). We can find no consistent differences between them. We therefore conclude that these should all be considered conspecific.

We therefore consider that *Hypolycaena dictaea* is not a separate species but is a subspecies (or possibly just a form) of *Hypolycaena phorbas* occurring on the islands of Waigeo, Misool and possibly Salawati, which all lie to the west of mainland New Guinea. We consider that the taxon present in Papua mainland, Aru, Roon, Biak and Yapen is *Hypolycaena phorbas silo*.

Batanta is a new distribution record for *Hypolycaena phorbas*. We have examined four males collected in October 2009 on the South Coast of the island. These all have the darker underside pattern. In the absence of females we prefer not to assign subspecific status.

We also make the following comments on Parsons’ suggested wider eastern distribution of *dictaea,* in the sense that he uses that name. The males from Waigeo, Misool, Batanta, Salawati, Papua mainland, Aru, Roon, Biak and Yapen, all localities within the western part of the species’ range, share the same shade of dark blue upperside. These contrast with the more purple colour of nominate *phorbas* and a number of un-named specimens in BMNH from the eastern islands of Papua New Guinea including Yule, Woodlark and Kiriwina (= Trobriand Islands).

In addition these more purple males have much darker undersides than the *dictaea* type. The origins of these un-named specimens match many of the localities given by Parsons included in his distribution of *dictaea*.

We believe he mistakenly included these together with Waigeo specimens in his provisional assessment of *dictaea*. As this latter, more purple, group is beyond the geographical scope of this article, we do not describe any of these specimens further, but await a more comprehensive revision of the genus. Nevertheless, the clarification herein of the status of the Maluku fauna should aid in such a revision.

***Hypolycaena sipylus***(Felder, 1860) (TL: Ambon)

*Hypolycaena sipylus* is widespread in Indonesia as well as occurring in the Philippines and New Guinea region (Rawlins 2007). It is the Type Species of *Hypolycaena*, although little is known of its early stages. In Maluku there are three recorded subspecific taxa.

**Figures 43-46.**
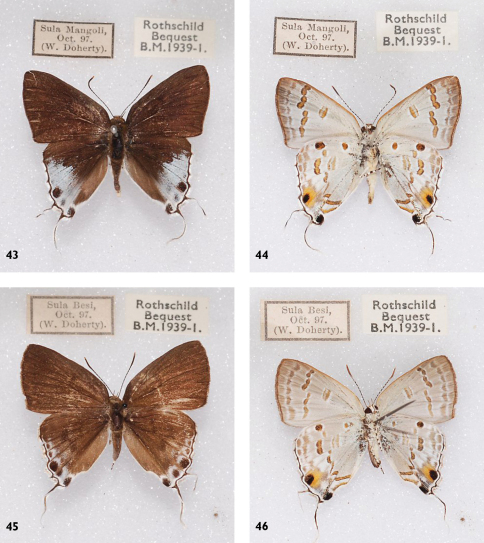
*Hypolycaena sipylus giscon*, l. to r.: Sula Islands, Mangole, ♂ Up, Un, Sula Islands, Sanana, ♀ Up, Un.

***Hypolycaena sipylus giscon*** Fruhstorfer, 1912 (TL: Sulawesi). Figs 43–46.

Within Maluku *Hypolycaena sipylus giscon* is known from Mangole and Sanana in the Sula Islands (Vane-Wright & de Jong, 2003). To this we add Taliabu (1♂ i/2005).

**Figures 47-50.**
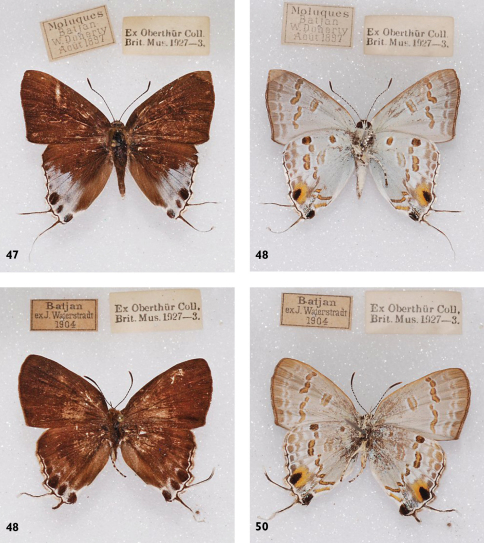
l. to r.: *Hypolycaena sipylus sipylus*, Bacan, l. to r.: ♂ Up, Un, ♀ Up, Un.

***Hypolycaena sipylus sipylus*** (Felder, 1860) (TL: Ambon). Figs 47–50.

The range of *Hypolycaena sipylus sipylus* is recorded by D’Abrera as “The Moluccas generally”. We have specific records from Morotai, Halmahera, Bacan and Obi in N. Maluku as well as Buru, Manipa, Kelang, Ambon, Seram and Kasa Island (off Seram) in C. Maluku.

**Figures 51-54.**
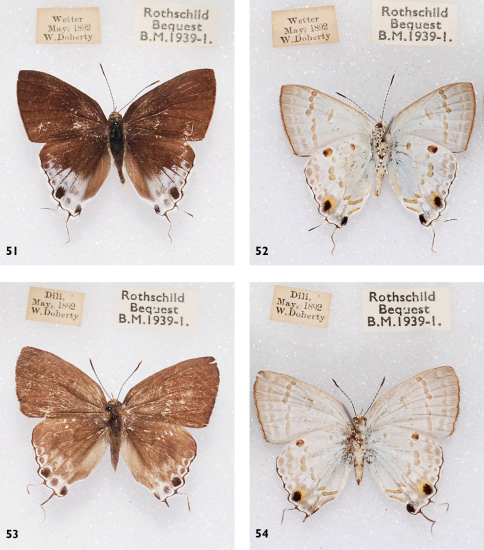
l. to r.: *Hypolycaena sipylus numa*, l. to r.: Wetar, ♂ Up, Un, Timor, Dili, ♀ Up, Un.

***Hypolycaena sipylus numa*** Fruhstorfer, [1912] (TL: Sumbawa, Flores). Figs 51–54.

*Hypolycaena sipylus numa* occurs on Wetar Island (Rawlins 2007) within S. W. Maluku as well as along the Lesser Sunda chain.

***Hypolycaena danis*** (C. & R. Felder, 1865) (TL: Halmahera)

This species occurs in Maluku Province in Indonesia as well as the New Guinea region and N. E. Australia. Fiedler (1992) points out that the morphology and biology of its early stages are nearly identical to those of *Hypolycaena othona* (Hewitson, 1865) and proposes an *othona* species group for those with elaborately camouflaged, orchid-feeding larvae with reduced myrmecophily.

**Figures 55-58.**
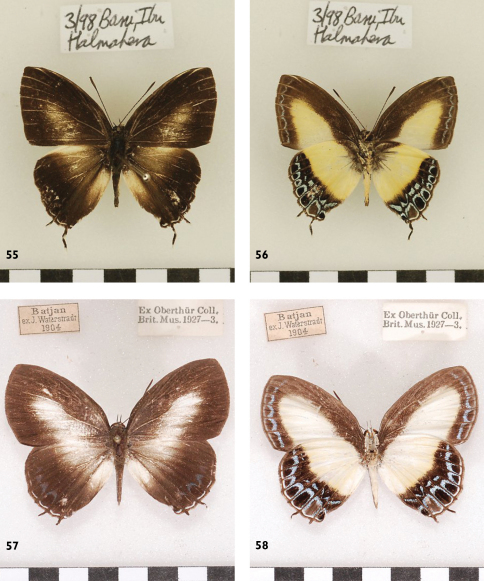
*Hypolycaena danis danis*, l. to r.: ♂ Halmahera, Up, Un, ♀ Bacan, Up, Un.

***Hypolycaena danis danis*** (C. & R. Felder, 1865) (TL: Halmahera). Figs 55–58.

= *Hypolycaena danis batjana* Fruhstorfer, [1916b] (TL: Bacan)

D’Abrera records *Hypolycaena danis danis* from Bacan and Halmahera in N. Maluku. To this we add Morotai (Daeo – 1♂ ii/1998, 1♀ v/2005, 1♀ vi/2005).

***Hypolycaena danis danisoides*** de Nicéville, 1897 (TL: “Key” Islands). Figs 59–70.

*Hypolycaena danis danisoides* occurs on the Kei Islands. BMNH has specimens from Little Kei (Kei Kecil) and to this we add Kei Besar (Yamtimur - 3♀♀ v/2002).

**Figures 63-66.**
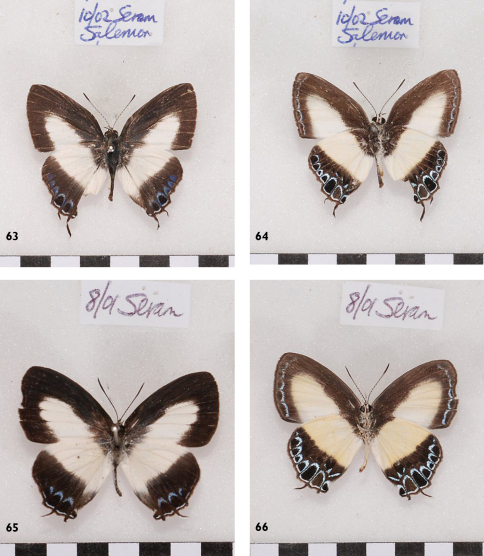
*Hypolycaena danis danisoides*, Seram, l. to r.: ♂ Up, Un, ♀ Up, Un.

Neither D’Abrera nor Parsons record *Hypolycaena danis* as occurring in C. Maluku but there are four males from Seram in BMNH. The second author has received several further specimens from Seram (1♂ and 1♀ vii/2001, 1♀ viii/2001, 1♀ vii/2002, 1♂ viii/2002, 2♂♂ and 1♀ x/2002, 1♀ x/2003, 1♀ vii/2004, 1♂ x/2007) and Ambon is added as a new locality record (Hila – 5♂♂ iv/2003, 1♀ ii/2008). The Seram specimens show a slight variation in phenotype of both males and females (Figs 63-66) but we include them within subspecies *danisoides.*

BMNH also has one male and three females from Obi which match this taxon. However there is a second male labelled Obi which is typical of the nominate subspecies from Halmahera. The specimen bears two labels:

1. “Obi, ex J. Waterstradt, 1904”.

2. “Ex Oberth Coll, Brit. Mus. 1927–3”.

Without further males to examine it is hard to draw a conclusion from this, but based on the other four Obi specimens we include these within *danisoides.* Therefore we extend the range of *Hypolycaena danis danisoides* to include Obi, Seram and Ambon as well as Kei.

**Figures 71-74.**
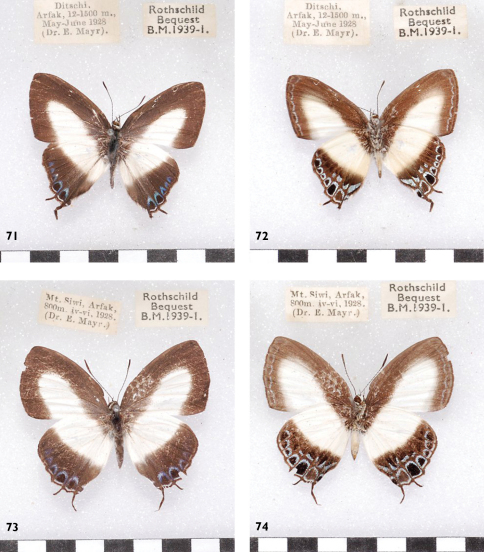
*Hypolycaena danis derpiha*, Papua mainland, Arfak, l. to r.: ♂ Up, Un, ♀ Up, Un. Similar to specimens to be found in Aru.

**Figures 75-79.**
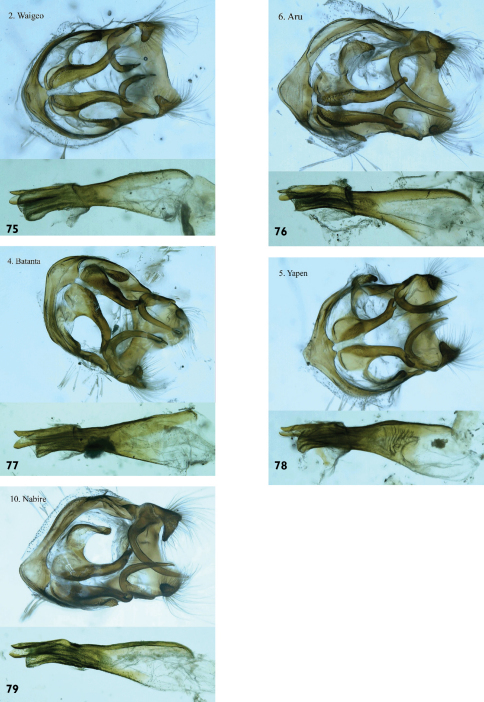
*Hypolycaena phorbas*, ♂ genitalia. Above: Armature in ventral or latero-ventral aspect. Below: Aedeagus in lateral aspect. **75.** *Hypolycaena phorbas dictaea* Waigeo Is. **76.** *Hypolycaena phorbas silo* Aru Is. **77.** *Hypolycaena phorbas silo* Batanta Is. **78.** *Hypolycaena phorbas silo* Yapen Is. **79.** *Hypolycaena phorbas silo* Nabire, Papua mainland.

***Hypolycaena danis derpiha*** (Hewitson, [1878]) (TL: Aru). Figs 71–74.

= *Hypolycaena danis deripha* [sic] D’Abrera, 1978.

D’Abrera records distribution as: “Aru (?) Papua and islands of Louisade Archipelago”. We assume he intends the “(?)” to refer to Aru, although Hewitson states the holotype to have been collected in Aru by Wallace. Although we could find no specimens from Aru in BMNH, M. Nagai (pers. comm.) says his son, K. Nagai, has collected three males and five females in Aru, confirming the type locality. This subspecies also occurs widely on the island of New Guinea including both Papua New Guinea (Parsons) and Papua mainland (Timika – 1♂ vi/2002, Nabire – 1♀ ii/2003, 1♀ iii/2003, Fak Fak – iv/2003).

## Discussion

Vane-Wright and Peggie (1994) conclude that the fauna of Central Maluku (Buru, Ambon, Seram, Seram Laut) is most strongly related to New Guinea and to Sulawesi plus the Philippines. The distribution of *Hypolycaena asahi* in Ambon and Seram, with two similar species in the Philippines, conforms to this pattern and supporting evidence for the theory. It also suggests that a closely-related species might occur in Sulawesi. *Hypolycaena umbrata* Seki and Takanami (1988) is a strong but not quite conclusive candidate. It shares with *asahi, shirozui* and *toshikoae* the larger hindwing tornal orange spot and deeply conjoined valvae, although the outer edges of the valvae are noticeably excavate with a sub-apical point. These four taxa might be shown in future to constitute the *shirozui* species group, but their monophyly is as yet uncertain.

Our extensive study of the *phorbas* species group taxa from Maluku, Papua Mainland and the islands of West Papua has clarified the status of the taxa *dictaea* and *erasmus*. This new information, when combined with further study of the related specimens from the islands to the East of Papua Mainland, should provide valuable evidence about the biogeography of the island arc from North Maluku to the East of Papua New Guinea and confirm the apparent monophyletic status of the species group.

## Conclusions

Examination of the male confirms the specific status of *Hypolycaena asahi* which is now recorded from Seram as well as the type locality Ambon. The species of *Hypolycaena* most closely resembling *asahi* occur in Sulawesi, Mindanao and Mindoro.

There is no confirmed record of *Hypolycaena periphorbas* (= *Hypolycaena erasmus*) occurring in Maluku. The distribution of this species remains extralimital, to the East of the region studied.

We synonymise *Hypolycaena erylus pigres* Fruhstorfer, [1912], with *Hypolycaena erylus thyrius* Fruhstorfer, [1912], the latter having page priority. The low number of specimens of *Hypolycaena erylus* available at this time from South and South East Maluku, especially of females, makes determination at subspecific rank for those islands speculative.

The p*horbas* species group sensu Fielder comprises *Hypolycaena phorbas*, *Hypolycaena erylus* and *Hypolycaena periphorbas*. *Hypolycaena dictaea* sensu Parsons, 1998, deserves at most subspecific rank and is restricted to certain islands to the west of Papua mainland.

## Summary of distribution of *Hypolycaena* species and subspecies in Maluku

**Table:** 

*Hypolycaena asahi*	C. Maluku: Ambon, Seram.
*Hypolycaena erylus gamatius*	Sula Islands: Taliabu, Mangole.
*Hypolycaena erylus thyrius*	N. Maluku: Morotai, Halmahera, Ternate, Bacan, Kasiruta, Obi.
*Hypolycaena erylus* ssp?	SE. Maluku: Tanimbar - Yamdena Island, Gorong Islands - Manawoka Island.
*Hypolycaena phorbas silo*	SE. Maluku: Aru.
*Hypolycaena sipylus giscon*	Sula Islands: Taliabu, Mangole, Sanana.
*Hypolycaena sipylus sipylus*	N. Maluku: Morotai, Halmahera, Bacan, Obi.
	C. Maluku: Buru, Manipa, Kelang, Ambon, Seram, Kasa Is.
*Hypolycaena sipylus numa*	SW. Maluku: Wetar.
*Hypolycaena danis danis*	N. Maluku: Morotai, Halmahera, Bacan.
*Hypolycaena danis danisoides*	N. Maluku: Obi; C. Maluku: Ambon, Seram; SE. Maluku: Kei.
*Hypolycaena danis derpiha*	SE. Maluku: Aru.

**Plate 1.**
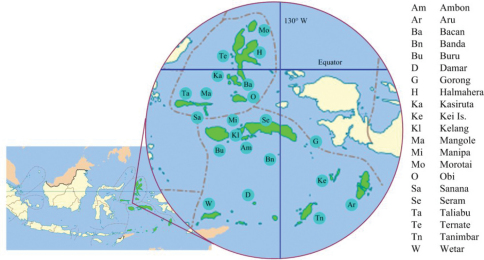
Islands of North Maluku and Maluku Provinces, shaded green, with key to named islands.

**Plate 2.**
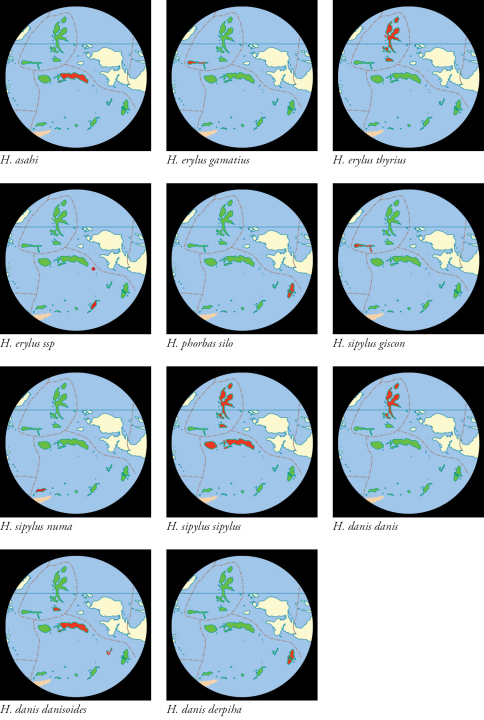
North Maluku and Maluku Provinces shown in green. Provincial boundaries chain dotted in red .Ranges of taxa shown in red.

## Figures and Tables

**Figures 39-42.**
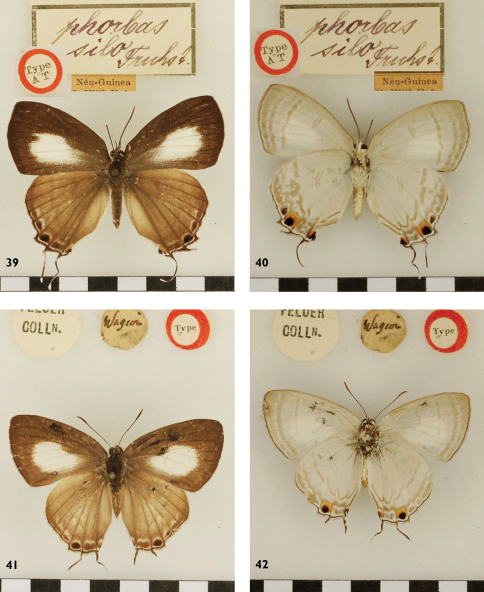
*Hypolycaena phorbas silo* ♀ Holoype Up, Un, New Guinea; *Hypolycaena phorbas dictaea* ♀ holotype, Up, Un, Waigeo.

**Figures 59-62.**
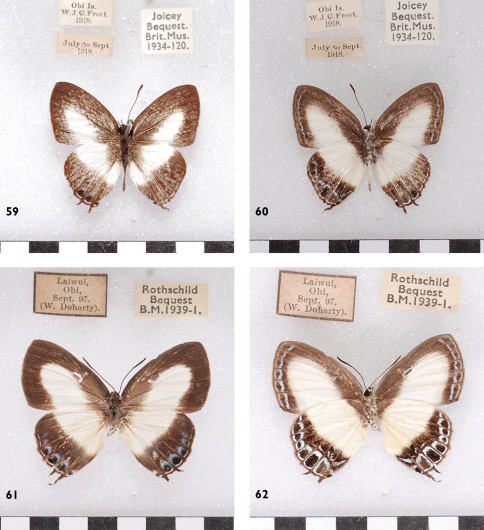
*Hypolycaena danis danisoides*, Obi, l. to r.: ♂ Up, Un, ♀ Up, Un.

**Figures 67-70.**
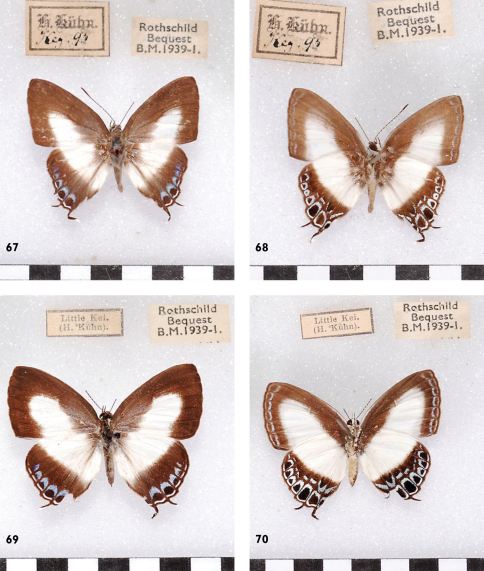
*Hypolycaena danis danisoides*, Kei, l. to r. ♂ Up, Un, ♀ Up, Un.
